# Supplementary material for: A Humanized Yeast Phenomic Model of Deoxycytidine Kinase to Predict Genetic Buffering of Nucleoside Analog Cytotoxicity
Source: Genes (Basel). 2019 Sep 30;10(10):770. doi: 10.3390/genes10100770 (PMC6826991; doi:10.3390/genes10100770)
Supplement: Supplementary file 1 [file genes-10-00770-s001.zip › Additional_Files/Additional_File7_GOTermHeatmaps.zip!/Additional_File7_GOTermHeatmaps/cohesin_complex.pdf]

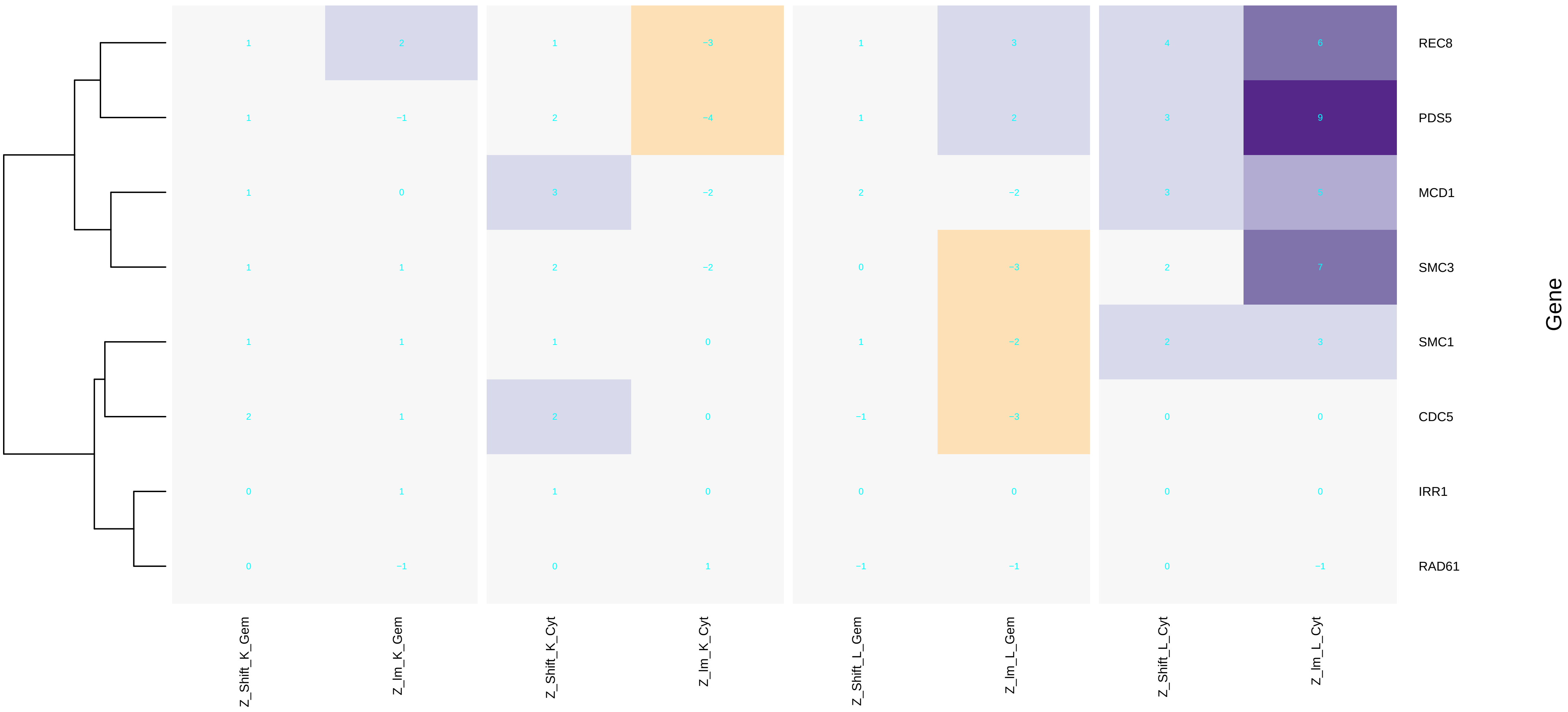

REC8 1 2 1 −3 1 3 4 6
PDS5 1 −1 2 −4 1 2 3 9
MCD1 1 0 3 −2 2 −2 3 5
SMC3 1 1 2 −2 0 −3 2 7
SMC1 1 1 1 0 1 −2 2 3
CDC5 2 1 2 0 −1 −3 0 0
IRR1 0 1 1 0 0 0 0 0
RAD61 0 −1 0 1 −1 −1 0 −1
Z_Shift_K_Gem
Z_Im_K_Gem
Z_Shift_K_Cyt
Z_Im_K_Cyt
Z_Shift_L_Gem
Z_Im_L_Gem
Z_Shift_L_Cyt
Z_Im_L_Cyt
Gene
